# The Blood Supply of Nerves

**Published:** 1891-04

**Authors:** 


### The Blood-Supply of Nerves.

At a recent meeting of the Academy of Sciences an interesting communication was made by MM. Quenu and Lejars on some new points in the vascular distribution in nerves. Having hit upon a novel and special method of injection, they were able to recognize in the circulatory apparatus of certain nerve trunks a series of constant dispositions hitherto but little, if at all, noticed. So far their investigations have been concerned with the cervical portions of the vagus and parts of the great sympathetic. They show that the recurrent laryngeals, together with the adjacent cervical portions of the pneumogastric and sympathetic, have their blood-supply from the thyroid arteries exclusively, and the authors suggest that herein may be found a ready explanation of the aphonia as well as the respiratory and vaso-motor modifications which are observed sometimes to follow thyroidectomy and ligature of the common carotid or thyroid arteries, and they deem it not unlikely that this fact of common blood-supply may be an element in the pathology of certain forms of Graves' disease. The veins of these nerves are more abundant than the arteries, and do not always play the part of satellites to these latter, but after forming a plexus on the ganglia of the sympathetic and pneumogastric, they empty themselves into either the network of the vasa vasorum of the common and internal carotids, creating thus an intimate connection between the wall of the artery and the nerve trunks which accompany it, or they join the thyroid veins, and especially a network of veins which covers the lateral wall of the pharynx; while others open into the veins in front of the vertebræ and into those of the pre-vertebral muscles. This connection of the veins of the nerve vascular system with muscular veins was found also to exist in the case of the limbs, and the authors submit that if it be allowed that muscular contraction is a factor in the circulation in small peripheral veins, it is evident that the anatomical peculiarity observed is calculated to aid favorably the expulsion of venous blood from the nerve trunks. The writers finally suggest that the abundant blood distribution in nerves may readily conduce to congestion, and that this, in its turn, may not be without importance in the pathology—as yet but little known—of neuralgia.—*Lancet.*

